# A global dataset of river network geometry

**DOI:** 10.1038/sdata.2018.127

**Published:** 2018-07-10

**Authors:** Emanuele Giachetta, Sean D. Willett

**Affiliations:** 1 Department of Earth Sciences, ETH Zürich, 8092, Zürich, Switzerland

**Keywords:** Climate change, Hydrology, Geomorphology, Tectonics

## Abstract

The plan-form structure of the world’s river basins contains extensive information regarding tectonic, paleo-geographic and paleo-climate conditions, but interpretation of this structure is complicated by the need to disentangle these processes from the autogenic behavior of fluvial processes. One method of interpreting this structure is by integrating channel length and drainage area as characterized by the scaling relationship between slope and area, resulting in a characteristic length parameter, referred to in recent studies as *χ*. In this paper, we apply this methodology at a continental scale by calculating *χ* for the world’s river networks. Mapping of *χ'*, a modified version of *χ* including the influence of precipitation distribution on river discharge and correction of base level for *χ'* in closed basins, illustrates the geometric structure of global river networks, thus highlighting where tectonics or changing climate have resulted in an apparent disequilibrium of the river channel geometry. Our global *χ* maps quantify a dynamic view of Earth’s river networks and help to identify past and ongoing evolution of Earth’s landscape.

## Background & Summary

Large-scale river networks, the backbone of most terrestrial landscapes, play an important role in the evolution of continental landmasses, ecosystems and human geography. The geometry of continental drainage networks results from the complex interactions between tectonic deformation, climate and substrate erodibility. Drainage networks evolve to efficiently remove water and sediment from the landscape, and their spatial pattern and the geometric characteristics of their branching networks reflect the tradeoff between tectonic uplift and deformation and geomorphic processes of erosion, all modulated by climate and rock type. The dynamics of these interactions imply motion of water divides and exchange of drainage area between basins through river capture. Drainage reorganization by river capture can significantly change the water discharge, sediment load and concentration of nutrients along river valleys, and alter the habitats for plant and animal species^1,2^. Given the importance of large drainage systems and their implications for biological evolution, catchment hydrology and geochemical processes, the plan-form geometry of river networks and their history of evolution have long been a topic of study, using a range of methodologies.

One method for analysing and comparing river networks is through the fundamental scaling relationships that exist in all tree-structure networks. For river channel networks, relationships exist between channel length, drainage area, branching frequency, and other geometric characteristics, but one of the most well-established and useful is the scaling between drainage area and channel slope, often referred to as Flint’s Law^3–8^. This relationship is empirical, but is consistent with a wide range of incision and sediment transport laws^9–12^, and can be given as

(1)S=ksA−θ ,

where *k*_*s*_ is referred to as the channel steepness, and *θ* is the channel concavity. Steepness is a function of the uplift rate and rock erodibility for many incision laws, and perfect slope-area scaling is expected only for equilibrium, i.e. steady-state river profiles. An integral form of this relationship can be obtained by integrating equation (1):

(2)z(x)=zb+A0−θksχ,

where *z*_b_ is the elevation at a base level, and *A*_0_ is a reference drainage area^13^. The integral term to the right is defined as a parameter, *χ*, and is an integral function of position in the channel network with dimensions of length:

(3)χ=∫xbx(A0A(x'))θdx',

where the integration is performed upstream from the base level to the location *x* along each channel^13^. At steady state, channels of a catchment underlain by uniform bedrock and experiencing uniform uplift, display linear *χ* versus *z* plots with a constant slope^13^.

The quantity *χ* can be calculated from digital topographic data and used as a valuable metric for drainage basin geometry. It provides a measure of the length of a channel or the elevation of a point in the channel, normalized for drainage area. The simplest interpretation of *χ* is that it serves as a proxy for steady-state elevation, under constant forcing conditions. Interpretation of profiles can yield important insights to transient behaviour of a geometrically stable network^13,14^.

*χ* can also be portrayed in map form^15^ in order to characterize the structure and geometry of a drainage basin. *χ* will increase systematically with distance from a river outlet with a rate of increase dependent on the network topology, geometry and area distribution. Perhaps most importantly is the ability to compare neighbouring drainage basins across divides. Channels in adjacent drainage basins must reach equivalent elevations in order to maintain continuity. For a basin or set of basins with constant, homogeneous uplift rate, rock erodibility and climate, *χ* and elevation scale linearly, so under these conditions, values of *χ* on opposite sides of a divide should also be equal. This must be true at all scales, from small interfluves to major divides separating large catchments. Contrasts in *χ* across water divides is thus an indication of transience in the basin geometry or of heterogeneity of uplift or erodibility parameters within the channel network. Contrasts in *χ* can arise from a number of sources, including differential base level fall, spatial variability in uplift or precipitation patterns, the exhumation of differentially-erodible rock, or rerouting of channels by sediment deposition. In all cases, the resulting transience alters river profiles and map patterns as channels reorganize through divide migration and river capture. Observations and analysis of the channel network through *χ* mapping can be an important part of recognition of landscape transience.

Differentiating between transience and spatial variability of relevant parameters is always a challenge in this analysis. Spatial variability in rock uplift rate, rock erodibility and precipitation rate can all contribute to equilibrium elevation in ways not captured by *χ* values and patterns and, in principle, should be accounted for^16^. Unfortunately, these are often difficult to estimate, even in the present. Of these, precipitation rate is the only one that is routinely estimated, so we bring this in the analysis. Willett *et al.*^15^, proposed a modified version of *χ* to include spatially variable precipitation rate:

(4)χ′=∫xbx(P0A0A(x′)P(x′))θdx'

where *P* is the mean annual precipitation rate at a given point in the drainage basin, and *P*_0_ is an arbitrary scaling factor for the precipitation rate^17^.

In this work, we combine two global datasets, one for topography, the other for mean annual precipitation in order to present global maps of *χ* and *χ*′ for the world’s river networks. The new world maps and seamless dataset allow users to investigate the dynamics of drainage networks, and to better understand and interpret the impact of drainage reorganization on the Earth system.

## Methods

Digital Elevation Models (DEMs) are fundamental data for many geoscience applications, and the accuracy and resolution of global elevation data have significantly improved during the last decade (e.g. GEMTED2010 (ref. 18), MERIT^19^). The construction of a *χ* map requires the calculation of hydrological attributes, e.g. flow direction, upstream drainage-area and river distances. As such, the sensitivity to the DEM is primarily though the ability to resolve accurate flow directions, channel connections and channel length. Accuracy of *χ* is not dependent on elevation accuracy as this comes into the calculations only through the hydrologic parameters. Information layers are usually obtained by applying routine Geographic Information System (GIS)-based methods to a DEM. The global HydroSHEDS^20^ and HYDRO1k^21^ datasets were used in this study because they provide ready-to-use, geo-referenced hydrographic information for regional and global-scale watershed analysis and modelling, and have been constructed to maximize the accuracy of hydrologic parameters. The *χ* maps of all continents are necessarily integrated from river outlets initiating from a common elevation (i.e., sea level). However, the representation of HydroSHEDS river networks is missing or truncated at latitudes north of 60° N, where data from the Shuttle Radar Topography Mission (SRTM) are not available^22^. Therefore, for drainage networks with outlets located south of 60° N, source data consist of the continental flow-direction grids available from the HydroSHEDS Version 1.2, at a resolution of 15 arc second (~500 m at the equator). For catchments with outlets located north of 60° N, data were taken from the HYDRO1k continental flow-direction grids, at a resolution of 30 arc second (~ 1 km at the equator). The portions of the HYDRO1k flow-direction grids overlapping the HydroSHEDS data were clipped to the boundaries of the HydroSHEDS drainage basins. As a result, the original delineation of HYDRO1k drainage basins and their river networks was altered along the clipped boundary. Errors in the clipped flow directions were visually detected by comparing the HYDRO1k-derived river networks with satellite images, and corrected manually.

### Extraction of hyodrological variables and construction of *χ* maps

Hydrological grid data were processed using the TopoToolbox-2 V. 2.0.1, a set of MATLAB functions that support the application of GIS methods in MATLAB^23,24^.

**Figure 1:**
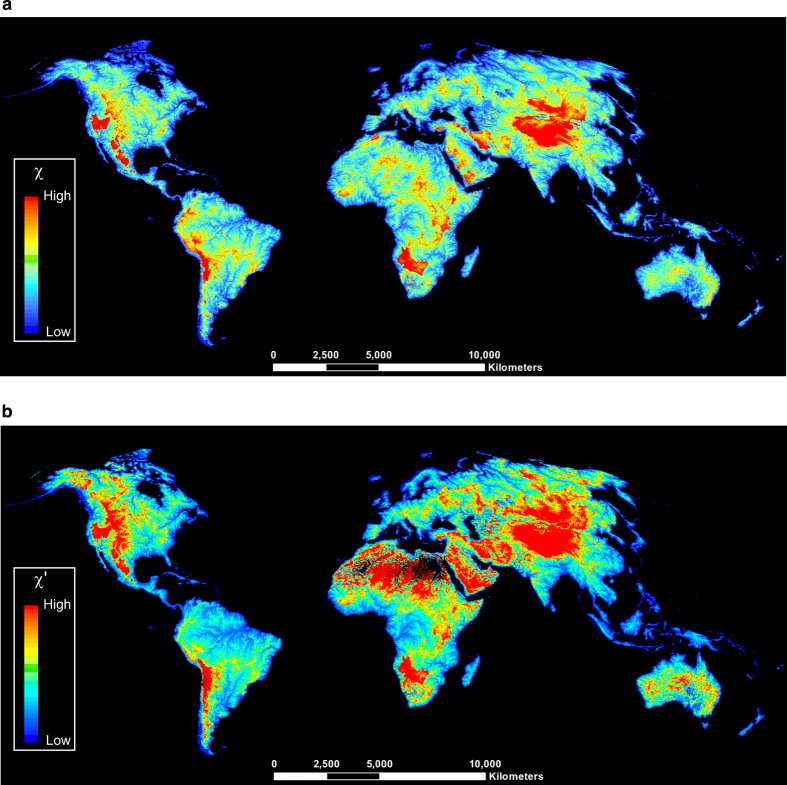
A global view of the dataset at 500 m and 1 km resolutions. (**a**) *χ* and (**b**) *χ′* maps for the World’s continents, excluding Antarctica and Greenland.

Each continental flow-direction grid of the source raster data was converted to ‘ASCII’ text files with ESRI ArcGIS version 10.5 software, and imported into MATLAB as ‘grid objects’ by using the ‘GRIDobj.m’ TopoToolbox-2 function^24^. Then each flow-direction grid object was converted to ‘flow objects’ by executing a custom version of the ‘FLOWobj.m’ TopoToolbox-2 function^24^. The HYDRO1k flow-direction grids for Europe, Asia and North America continents were imported in TopoToolbox-2 in their native projections and grid resolution of 1 km. To avoid degradation of pixel values in the continental flow-direction grids during reprojection^25^, the HydroSHEDS source data were imported into MATLAB in their native spherical coordinate system (i.e. latitude-longitude), which is based on the WGS84 datum. The surface area of each latitude-longitude quadrangle corresponding to a pixel in the continental HydroSHEDS grids was converted to square meters by using the ‘areaquad.m’ MATLAB function, with the latitude of the parallels and the longitude of the meridians bounding each pixel area and the WGS84 ellipsoid as input variables. As a result, we obtained a ‘pixel area’ grid from each continental HydroSHEDS flow-direction grid. River networks were produced by calculating the accumulated flow as the number of all cells flowing into each downslope cell by using the ‘flowacc.m’ TopoToolbox-2 function^24^. The continental flow-accumulation grids were obtained from each flow-direction grid, which is used to determine the downslope flow direction for each cell. For the HydroSHEDS grids, the ‘pixel area’ grids were used as a weight raster to calculate accumulated flow due to spatially variable pixel area. The scaling of river slope and drainage area of equation (1) is established only for drainage area larger than a critical area, typically *A*_c_>1 km^2^ (ref. 26), or even smaller. However, at a global scale, a critical area of this size produces nearly intractable data file sizes. Computationally this is not an issue, but visualization remains an issue, so we made all computations using a critical drainage area *A*_c_=5 km^2^. A logical grid, which indicates the location of river pixels in flow-accumulation grids, was obtained from each flow-accumulation grid using the threshold value *A*_c_. River networks and upstream flow-distances were calculated as ‘stream objects’ by passing the flow-direction and channel-network location grids as input variables to the ‘STREAMobj.m’ TopoToolbox-2 function^24^. Each ‘stream-object’ includes information on geometry and topology of the stream-network derived from a ‘flow object', as well as properties of the stream network (e.g. distance of river pixel from the outlet). For the HydroSHEDS river networks, the length of each channel segment was converted to meters by using the ‘distance.m’ MATLAB function, which calculates the length of the great circle arc connecting pairs of points with latitude-longitude coordinates on the surface of the standard WGS84 ellipsoid. The continental *χ* maps were calculated following the protocol described by Willett *et al.*^15^ and Yang *et al.*^17^, by setting the river concavity *θ* to a reference value of 0.45 in equation (3)^27^, a scaling area *A*_0_=1 m^2^ (ref. 15), and calculating the integral of equation (3) starting from each outlet and ending at the channel head of each river. Integration of the term inside brackets of equation (3) was performed with the ‘Trapezoidal’ method. Each continental river network was converted into MATLAB structure arrays, where the values of the continental *χ* map grids were stored by executing the ‘STREAMobj2mapstruct.m’ TopoToolbox-2 function. Eventually, we utilized the ‘shapewrite.m’ MATLAB function to convert each *χ* map structure array into an ESRI shapefile (Fig. 1a). Each continent *χ* map layer was then split into 10° x 10° tiles with a geographic projection referenced to the WGS84 datum (Data Citation 1).

### Correction of *χ* for closed basins

Maps of *χ* are constructed initiating integration at sea level. Internally drained basins present a problem in this analysis, in that the integral must be initiated at the lake, inland sea or other internal drainage sink that has a base level other than sea level. In order to compare *χ* values from basins with differing base level, the *χ* integration requires an initial *χ* value as well as an initial elevation value. In order to obtain initial values for non-sea-level basins, we made an estimate of the global steepness. From the global HYDRO1k DEM, we calculated a global channel steepness *k*_*s*g_=32.2, where this is the slope value of *k*_s_ from equation (1) by using the ‘gradient8.m’ Topotoolbox-2 function^24^ to produce a global channel slope grid. Subsequently, we extracted the elevation *z*_i_ of the lowest point in each closed basin *i* and calculated the starting value for the integration as:

(5)χcorrcbi=zi/ksg.

Closed basin polygons were extracted from the ‘Drainage Basins’ vector layers of HYDRO1k and HydroSHEDS. All pixels of closed basins polygons were used as masks to locate all pixels of closed basins on each continental *χ* map grid. Then, we added the quantity *χ*_corrcbi_ to all pixels of closed basins (Data Citation 1).

### Precipitation corrected *χ’* maps

**Figure 2:**
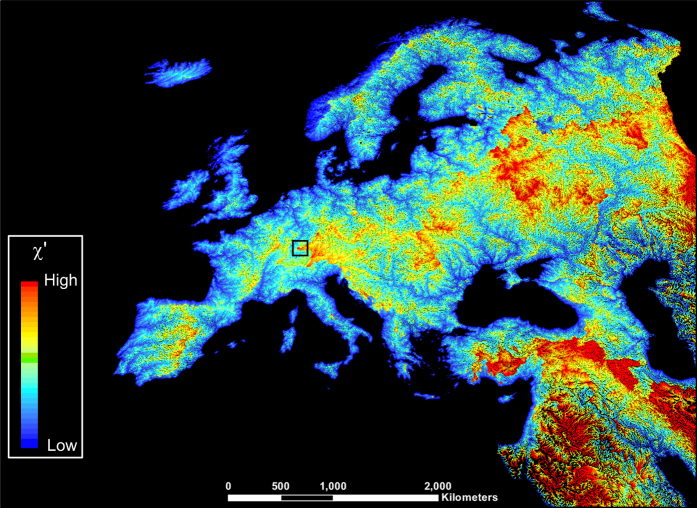
A view of the output data for drainages of the European continent. Map of *χ'* for Europe; disequilibrium in drainage networks is evident in the central part of the continent, where adjacent channel heads display high color contrast across the intervening water divide (yellow to red).

We included the influence of the spatial variation of precipitation across continental landmasses in the calculation of *χ'* as defined by equation (4). Continental precipitation grids were extracted from the global monthly precipitation grids at 30 arc second spatial resolution (~ 1 km at the Equator), averaged over the period 1960–1990, and available from the WorldClim dataset^28^. The average annual global precipitation grid was calculated by summing the 12 monthly precipitation grids. Then, the original global precipitation grid was resampled to match the resolution of 15 arc second HydroSHEDS grid. The gridded values of global precipitation were then utilized as weights in the calculation of the flow-accumulation grids. For HYDRO1k river networks, the calculation of precipitation-weighted flow accumulation was obtained by using precipitation and flow-direction grids as input variables to the ‘flowacc.m’ TopoToolbox-2 function. For HydroSHEDS river networks, precipitation-weighted flow accumulation was obtained by passing the flow-direction and both the precipitation and the ‘pixel area’ grids as weights to the ‘flowacc.m’ function. River pixels were located on the flow-accumulation grids using the threshold value *A*_c_. River networks and upstream flow-distances were obtained by using the ‘STREAMobj.m’ TopoToolbox-2 function. To calculate *χ'* maps from equation (4), we implemented a scaling precipitation rate *P*_0_=1 my^−1^ and a scaling area *A*_0_=1 m^2^ (ref. 17). The values of *χ'* were corrected for closed basins as previously described, and then each continental *χ'* map grid was converted to ESRI shapefile using the function ‘shapewrite.m’ in MATLAB (Figs. 1b and 2). Each continent *χ'* maps layer was then split into 10°×10° tiles with a geographic projection referenced to the WGS84 datum (Data Citation 1).

### Simplified *χ* and *χ'* maps

**Figure 3:**
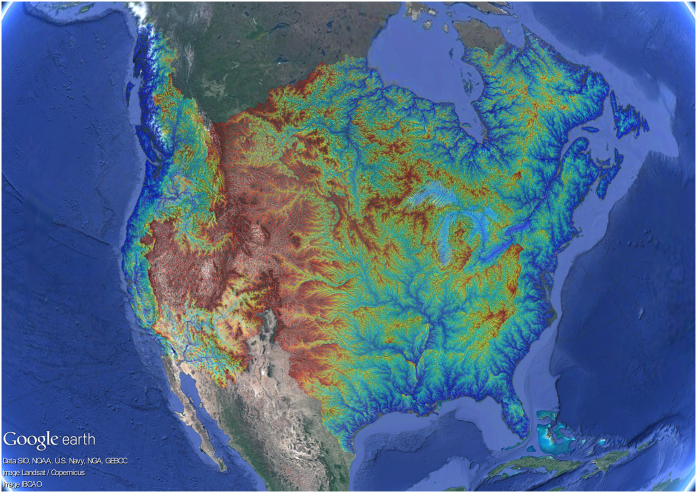
Simplified map of *χ'* for the North America continent. Each simplified *χ'* map was converted to .kml format for importing and viewing data in free applications like Google Earth.

Analysis of river network stability is usually performed along drainage divides by comparing *χ* at channel heads at opposing positions across a water divide^15^. Reducing the number of river network segments can facilitate the conversion of each continental *χ* and *χ'* map into a .kml file of reasonable size for importing and viewing in free applications like Google Earth. For this purpose, we constructed ‘simplified’ maps of *χ* and *χ'* by deleting many nodes, retaining only nodes at 1) confluences, 2) outlets and 3) channel heads from the original river networks. This strategy maintains the drainage network topology while reducing the size of each continental shapefile. This results in visual degradation of the channel network, with long linear segments that appear to cross topography, but the values of *χ* and *χ'*, and the positions of channel ends and confluences retain the resolution of the original calculation. Each simplified *χ* and *χ'* map was converted to a .kmz (Keyhole Markup Language Zipped) file by implementing a custom MATLAB code (Data Citation 1; Fig. 3).

### Code availability

The MATLAB software package of TopoToolbox-2 (ref. 24) is available at http://www.earth-surf-dynam.net/2/1/2014/esurf-2-1-2014-supplement.zip. The custom codes for data processing, developed using MATLAB R2015b software in the Windows 7 environment, are available from the corresponding author upon request.

ESRI ArcGIS Desktop version 10.5 software was utilized in a Windows 7, 64 bit operating system to produce the output datasets.

## Data Records

The ‘Global *χ* Maps Dataset’ (Data Citation 1) consists of ready to use vector data layers in shapefile and .kmz formats, representing global coverage maps of *χ* and *χ'* described in the Methods section. The shapefiles are provided in geographic projection referenced to the WGS84 horizontal datum, and are publicly available and freely accessed through figshare (Data Citation 1). Each set of spatial data is obtained by downloading the corresponding .zip archive file (Table 1). The spatial data shared in these archives can be employed for the analysis of the plan-form geometry of drainage networks at continental scale.

## Technical Validation

Uncertainty in the output data stems from the errors in the input data, whose quality has been assessed by previous studies^29–31^. The accuracy of river network representation in HydroSHEDS significantly exceeds that of HYDRO1k and other existing global datasets, though it has not been evaluated systematically^31^. For calculation of *χ*, only the flow direction, network topology, contributing area, and channel length are important. The HydroSHEDS flow directions are more susceptible to errors in regions of varying vegetation cover, water bodies and flat regions with poorly defined elevation. Errors in the river network delineation are also due to upscaling of river networks from 3 to 15 arc second resolution, and applying a single drainage direction algorithm^31^.

The accuracy of the output data cannot be thoroughly assessed due to the lack of similar datasets. The concavity, *θ*, typically has a ratio of about 0.45 and we have used this nominal value to construct the global *χ* and *χ'* maps. This value may not accurately reflect the average steady-state channel concavity for all the catchments mapped, and is a limitation of any global analysis, such as presented here. We expect that the resolution of the source data affects the accuracy of our channel length and drainage area calculation^25,32^, although this impact has not yet been evaluated. In particular, channel length is underestimated for rivers with sinuosity higher than can be resolved by the cell size.

## Usage Notes

### Dataset Organization

The output datasets are included in the .zip archive files:

1. ‘Global_Chimaps_shp_Data_Record_A’, including: a) ten full-resolution ESRI ArcGIS shapefiles representing a continent or subcontinent *χ* map, b) the symbology that we used to represent vectorial *χ* map data in Fig. 1a, stored in an ArcGIS Layer file in .lyr format and in a Styled Layer Descriptor (SLD) file in XML format, and c) 360 10°×10° tiles of continental *χ* maps supplied in a subfolder along with a global map in .pdf format showing number and location of each tile;
2. ‘Global_Chipmaps_shp_Data_Record_B’, including a) ten ESRI ArcGIS shapefiles representing a continent or subcontinent *χ'* map, b) the symbology that we used to represent vectorial *χ'* map data in Fig. 1b, stored in an ArcGIS Layer file in .lyr format and in a Styled Layer Descriptor (SLD) file in XML format, and c) 356 10°×10° tiles of continental *χ'* maps supplied in a subfolder along with a global map in .pdf format showing number and location of each tile;
3. ‘Global_Simplified_Chimaps_kmz_Data_Record_C’, including 10 subfolders; each folder contains spatial data in .kmz format, representing a subset of each simplified continental *χ* map extracted from the continental shapefiles in Data Record A. The .kmz files can be viewed in free software like Google Earth. To orient the user, a Google Earth view of each subset of *χ* map is attached in .pdf format to the corresponding .kmz file.
4. ‘Global_Simplified_Chipmaps_kmz_Data_Record_D’, including 10 subfolders; each folder contains spatial data in .kmz format, representing a subset of each simplified continental *χ'* map extracted from the continental shapefiles in Data Record B. The .kmz files can be viewed in free software like Google Earth. To orient the user, a Google Earth view of each subset of *χ'* map is attached in .pdf format to the corresponding .kmz file.
5. Metadata is stored in an ISO-format XML file.

### Naming Conventions

The name of each shapefile starts with two or three letters that are abbreviations of a continent or subcontinent name:

- ‘Af’ = Africa

- ‘As' = Asia

- ‘Au’ = Australia

- ‘CAm’ = Central America

- ‘Eu’ = Europe

- ‘NAm’ = North America

- ‘SAm’ = South America

Two shapefiles are archived for Europe, North America, and Asia continents:

1. the ones named ‘..._15s_...’ include data records for drainage basins with outlet located south of 60° N derived from the HYDROSHEDS flow direction grid, at a resolution of ~500 m;
2. the ones named ‘..._1k_...’ include data records for drainage networks with outlet located north of 60° N derived from the HYDRO1k flow direction grid, at a resolution of ~1 km.

To facilitate the user's orientation, the data records in .kmz format containing subsets of each simplified continental *χ* and *χ'* map are archived in different folders, whose name ends with one or two letters that are initials of the cardinal directions:

- ‘N’ = North

- ‘S’ = South

- ‘E' = East

- ‘W’ = West'.

A second letter, ‘C’ standing for ‘central', is used to further identify a subset of spatial data extracted from very large continental maps.

### Confirmation from elevation

**Figure 4:**
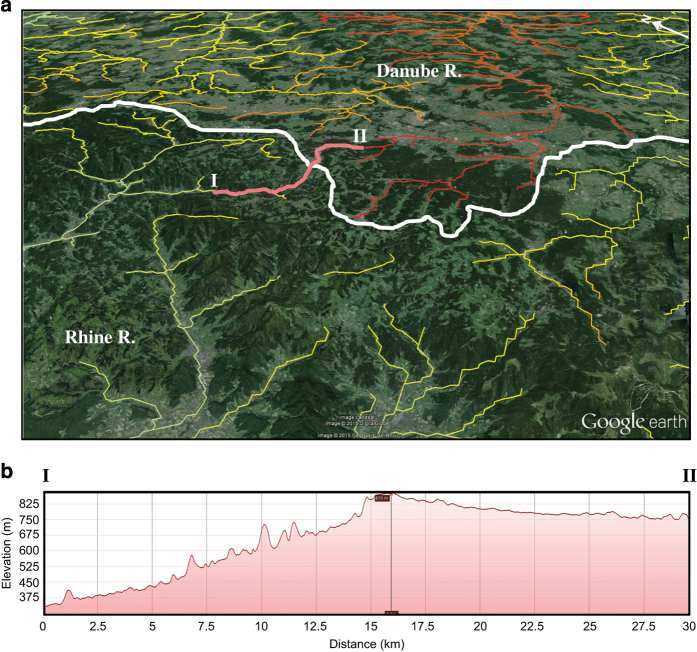
An example of geometric disequilibrium interpreted from the *χ* map and confirmed through topography. (**a**) The high *χ*’ values in the upper Danube catchment are juxtaposed with lower maximum values in the adjacent Rhine catchment. White line in (**a**) represents the main drainage divide between the Danube and Rhine drainage basins. Pink line in (**a**) represents a map view of the topographic profile across the main divide that is shown in (**b**). (**b**) Topographic profile approximately following the valley bottoms from a tributary of the Rhine into the Danube catchment. Asymmetry in the topographic profile is consistent with the contrast in the *χ* map and supports an interpretation that the water divide is migrating towards the Danube catchment.

Given that *χ* and elevation scale at steady state, the resulting *χ* and *χ'* maps allow the interpretation of equilibrium in drainage basins on opposite sides of divides. Other than determining direction of water flow, these maps do not use elevation data, which can therefore be used as an independent confirmation of drainage stability. Confirmation of river network stability can be checked by comparing *χ* or *χ'* maps with topographic asymmetry across drainage divides. Examples are provided in Fig. 4a and b using Google Earth topography.

## Additional information

**How to cite this article**: Giachetta, E. & Willett, S. D. A global dataset of river network geometry. *Sci. Data* 5:180127 doi: 10.1038/sdata.2018.127 (2018).

**Publisher’s note**: Springer Nature remains neutral with regard to jurisdictional claims in published maps and institutional affiliations.

## Supplementary Material



## Figures and Tables

**Table 1:** Output ‘Global *χ* Maps Dataset’ archives.

Name	Format	Description
**Global_Chimaps_shp_Data_Record_A.zip**	shapefile	*χ* maps corrected for closed basins
**Global_Chipmaps_shp_Data_Record_B.zip**	shapefile	*χ'* maps corrected for closed basins and precipitation
**Global_Simplified_Chimaps_kmz_Data_Record_C.zip**	.kmz	Simplified *χ* maps corrected for closed basins
**Global_Simplified_Chipmaps_kmz_Data_Record_D.zip**	.kmz	Simplified *χ'* maps corrected for closed basins and precipitation
Name of data record, format and description of vector datasets included in each .zip archive.		
